# The Role of Negative Pressure Wound Therapy (NPWT) in the Management of Vasculitic Wounds: Case Series of Eight Patients

**DOI:** 10.1177/15347346211063700

**Published:** 2021-12-08

**Authors:** Kirsi Isoherranen, Nicolas Kluger, Katariina Hannula-Jouppi, Liisa Väkevä

**Affiliations:** 159841 Department of Dermatology and allergology, University of Helsinki and Inflammation center, Helsinki University Hospital, Helsinki, Finland

**Keywords:** Inflammatory ulcer, atypical ulcer, cutaneous vasculitis, negative pressure wound therapy

## Abstract

Vasculitic ulcers belong to the category of atypical ulcers and are traditionally very slow to heal. The aim of this study is to retrospectively analyze the files of eight patients with vasculitic ulcers treated with negative pressure wound therapy **(**NPWT). Immunosuppression was initiated at least two weeks prior to starting NPWT. We suggest that this is a safe and promising protocol to treat these hard-to-heal ulcers.

## Introduction

Cutaneous vasculitis is defined as an inflammatory reaction of the vessel wall leading to vascular damage. Recently, a new nomenclature of cutaneous vasculitis has been published, that defines the systemic and cutaneous variants of medium-vessel and small vessel vasculitides.^1,2^ Cutaneous vasculitis typically has an acute course and immediate intervention therapy is needed to avoid further damage. Various immunosuppressive therapies are the cornerstone of treatment. Usually, high-dose systemic corticosteroid treatment is used to cope with acute vasculitis but long-term corticosteroid treatment has multiple side effects.^3^ Therefore, it is necessary to consider other treatment modalities with less systemic side-effects to curb the inflammatory reaction. Alternative treatment modalities include eg methotrexate, azathioprine, cyclosporine, mycophenolate mofetil, cyclophosphamide, and rituximab; and the treatment is modified according to the vasculitis type.^2,4^

Normal wound healing is a complex biological process including interaction with different cell types and biochemical signals.^5^ Chronic wounds typically stagnate at the inflammatory phase, where wound healing is characterized by the release of cytokines and inflammatory proteins such as TNF-α, IL-1, and TGF-β. Neutrophils are the first cells to invade and clean the area by releasing proteolytic enzymes as well as matrix metalloproteinases (MMPs). The influx and activation of tissue-resident macrophages are also important in the wound healing process. They produce growth factors eg platelet-derived growth factor (PDGF), inflammatory proteins (TNF-α, IL-6), allure fibroblasts and secrete different enzymes, which degrade deteriorated tissue. Little is known about the inflammatory process in vasculitic wounds, but probably MMPs play a role.^6,7^

Negative pressure wound therapy (NPWT) has shown great advantages in the management of a wide range of wounds,^8^ but reports of its use in inflammatory wounds are scarce.^9,10^ The mode of action of NPWT is based on distinct mechanisms which support and improve the wound healing environment. In traumatic wounds, NPWT increases local vascular endothelial-derived growth factor (VEGF) expression in humans thus promoting angiogenesis and vascular proliferation.^11,12^ In animal models, it has shown to increase the accumulation of granulation tissue.^13^ NPWT also increases blood flow in ulcers and promotes wound contraction.^14^ The expression of MMP-2 and MMP-9 are increased in chronic wounds^15^ and NPWT has shown to reduce the expression of MMP-9.^16^

Previous reports of NWPT treatment in dermatologic conditions include eg pyoderma gangrenosum, hidradenitis suppurativa, and Bechet disease.^9,10,17–20^ However, we are not aware of previous studies or case series about NWPT treatment of vasculitic ulcer (VU) patients although NWPT is commonly used in wound management nowadays. In this study, we describe the effectiveness of NWPT in VU patients.

## Patients and Methods

We retrospectively analyzed the files of eight VU patients treated with NPWT from 2012 to 2019 at the Helsinki University Hospital. Patient gender, age, type of vasculitis, immunosuppression used, and duration of NPWT treatment was recorded (Table 1). The result of NPWT treatment was analyzed from the patient files and was qualitative in nature. In addition, we describe in detail one of the patients, a 10-year-old girl with VU who was successfully treated with NPWT.

**Table 1.** Case Series of Eight Patients with Vasculitis Ulcers Treated with NPWT.

	Sex	Age years	Diagnosis	Immunosuppressive treatment	Immunosuppressive treatment before NPWT (weeks)	NPWT duration (weeks)	Improved granulation	Reduction of wound size	Skin grafting	Complete healing	Treatment failure
1	F	71	MCTD, leucocytoclastic vasculitis	Prednisolone 35 mg/day + methotrexate 10 mg/week	4	12	x	x	x	x	
2	M	77	Leucocytoclastic vasculitis	Prednisolone 55 mg/day	4	8	x	x	x	x	
3	F	75	Leucocytoclastic vasculitis	Prednisolone 40 mg/day	2	2	x				x (activation of vasculitis)
4	F	69	Leucocytoclastic vasculitis	Prednisolone 40 mg/day	104	1					x (eczema in periwound skin)
5	F	77	CREST, leucocytoclastic vasculitis	Prednisolone 40 mg/day + azathiopurine 100 mg/day	20	4	x	x			
6	F	10	STING-associated interferonopathy	Prednisolone 25 mg/day + cyclosporine 50 to 100 mg/day	2	2	x	x		x	
7	F	68	Leucocytoclastic vasculitis	Prednisolone 60 mg/day, cyclosporine 50 mg/day, methotrexate 25 mg/week	Several years	6	x			x	
8	M	68	Leucocytoclastic vasculitis	Prednisolone 40 mg/day, methotrexate 7,5 mg/week	8	12	x		x	x	

### Case

**Figure 1.**
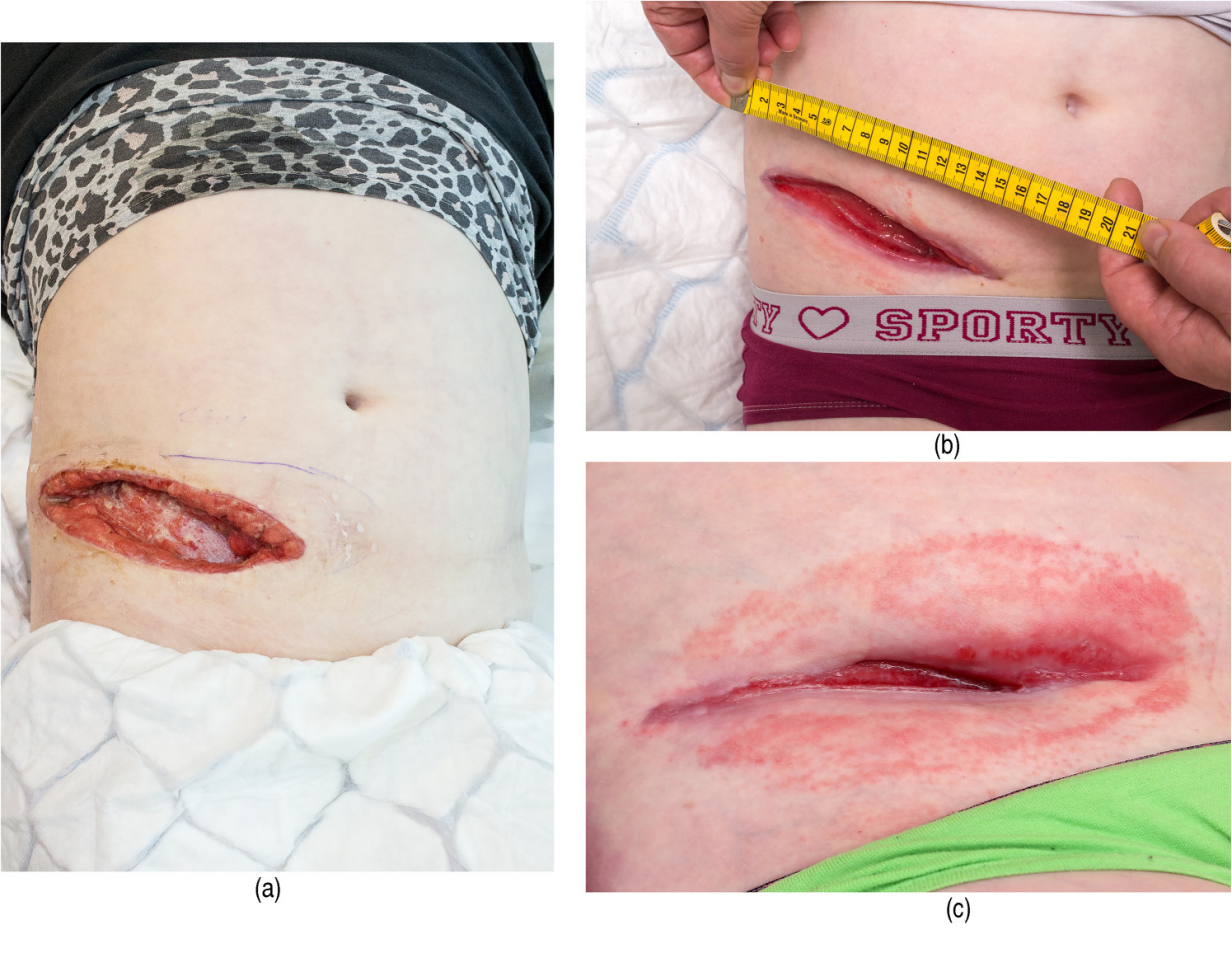
(a) The wound of a 10 year old girl suffering from STING- associated vasculopathy with onset in infancy (SAVI) before NPWT treatment. (b) The wound after four weeks of NPWT therapy. (c) The wound after six weeks of NPWT therapy.

A 10-year-old girl was diagnosed with a stimulator of interferon genes (STING) p.Gly207Glu mutation leading to a rare interferonopathy causing several lupus-like features and features of STING- associated vasculopathy with onset in infancy (SAVI).^21^ She had livedo reticularis on the trunk, arms, and legs since birth. She initially developed a small pustule/violaceous lesion on the right abdomen, with increasing redness, and was prescribed cephalosporine orally. Within a few days, she developed a fever and was admitted to the pediatric surgical emergency department. Her CRP level was elevated to 254 mg/L (N < 3) and after surgical revision of the necrotic lesion, clindamycin was started intravenously. A revision was made up to the fascia, which appeared clinically clear. Bacterial culture revealed *Streptococcus pyogenes* and *Staphylococcus aureus.* Three days postoperatively the wound began to enlarge and redevelop necrosis. After a dermatology consultation, a biopsy was taken and oral prednisolone was initiated at 25 mg/day (0.5 mg/kg/d). The biopsy revealed thrombosis, destroyed vessels with neutrophil accumulation, and extravasation of red blood cells indicative of vasculitis. Gram-staining was negative. Further surgical revisions and closure were avoided at this stage due to active vasculitis. After two weeks, no significant healing was seen and cyclosporine was started at 25 mg twice daily (1.05 mg/kg/d) and two weeks after, the dose was adjusted to 50 mg twice daily (2.1 mg/kg/d). Prednisolone was continued initially at a dose of 20 mg/day and tapered down to 10 mg/day when the cyclosporine dose was raised. NPWT was commenced in order to accelerate wound healing two weeks after starting prednisolone (Figure 1a). Momethasone liniment was applied on the surrounding skin below the NPWT dressings to avoid irritation. NPWT was painless and well-tolerated. After four weeks, there was a clear diminishment of the wound and improved granulation tissue (Figure 1b). After six weeks (Figure 1c), there was some irritation in the peri-wound area, and as the wound was very small and well granulated, it was decided to discontinue NPWT and continue with traditional wound dressings. Nine weeks after the initiation of NPWT, secondary closure was performed and the wound was closed completely. During a three-year follow-up, there were no wound recurrences.

## Results

We collected data from eight patients, two males and six females (Table 1). Leucocytoclastic vasculitis was found in majority (7/8). The duration of immunosuppression before NPWT treatment varied from two weeks to several years. The medications used included prednisolone in all, combined with azathioprine, methotrexate, or cyclosporine as the steroid-sparing agents in five patients. The duration of NPWT treatment varied from one to twelve weeks with an average treatment period of 6.6 weeks. One patient with only prednisolone at 40 mg/d as an immunosuppressant for two weeks prior to NPWT had activation of vasculitis on the wound edges, and NPWT treatment was discontinued after two weeks. One patient had eczema on the peri-wound skin and NPWT treatment was discontinued after one week, but with the other patients, there were no problems with the peri-wound skin. In 7/8 patients NPWT treatment improved the granulation and diminished the wound size (Figures 1a,b,c) and led to complete closure in three patients. In three patients, skin grafting was performed after NPWT treatment with partial or complete healing (Table 1). During one year follow-up, there were no wound recurrences.

## Discussion

Typically, VUs heal very slowly and patients are treated for long periods at dermatological wards and outpatient clinics. During the active phase of vasculitis, revision and surgical treatment of wounds can lead to increased necrosis and enlargement of wounds due to the pathergy phenomenon. Therefore, treatment of VU has been very conservative to date, relying mainly on immunosuppressive treatment.^4^

However, after the active inflammation is reduced with immunosuppressive medication, there is no reason why more modern wound treatments could not be used. NPWT has become an effective tool in the treatment of complex wounds.^17,22,23^ Recently, it has also been reported, that NPWT and skin grafting is a good option for treating Pyoderma gangrenosum wounds ^15^

In chronic wounds, the healing process stagnates at the inflammatory phase. In VU, inflammation itself prevents the normal healing process. High-dose corticosteroid treatment is used in the acute phase, but based on our results NPWT can be safely used approximately two to four weeks after the initiation of immunosuppressive treatment to prevent the prolongation of the ulcerative phase. Interestingly, NPWT reduces the expression of MMP-9, which in humans is expressed in leucocytoclastic vasculitis.^6^ MMP-9 enables vessel wall invasion by monocytes in giant cell arteritis^7^, thus the reduction of MMP-9 by NPWT might play a crucial role in VU healing.

This retrospective analysis showed that NPWT is a safe and effective treatment option in VU, as long as immunosuppressive treatment had decreased the vasculitis activity before NPWT. The shortest time scale from the beginning of immunosuppressive treatment to the beginning of NPWT was 2 weeks in our material. The duration of NPWT in these patients varied between 4 to 12 weeks. VUs are typically very slow to heal and correspondingly also the duration of NPWT was quite long. Skin grafting was performed in three patients after NPWT with good results, similar to observations in pyoderma gangrenosum wounds.^15^ The average duration of NPWT in our patients was 6.6 weeks. Histological studies have shown that optimal wound bed quality is achieved in 30 to 45 days of NPWT treatment, so our results are consistent with this finding.^24^

Our patient population was rather heterogeneous, and our study was retrospective by nature. Thus, further prospective studies are needed in order to define more clearly the optimal timing and duration of NPWT treatment in VU. However, we suggest that NPWT is a good option in treating VU after immunosuppressive treatment has controlled the inflammation in the wound area. According to our clinical experience, peri ulcer skin irritation and activation of vasculitis can also be suppressed by using corticosteroid liniment under NPWT dressings. We suggest that NPWT also leads to faster wound healing in VU as in chronic ulcers.

## References

[1] Sunderkötter CH Zelger B Chen KR , et al. Nomenclature of cutaneous vasculitis: dermatologic addendum to the 2012 revised international chapel hill consensus conference nomenclature of vasculitides. Arthritis Rheumatol. 2018 Feb;70(2):171-184. 10.1002/art.40375 29136340

[2] Isoherranen K ÒBrien JJ Barker J , et al. Atypical wounds. Best clinical practice and challenges. J Wound Care. 2019 Jun 1;28(Sup6):S1-S92. 10.12968/jowc.2019.28.Sup6.S1 31169055

[3] Hartmann K Koenen M Schauer S , et al. Molecular actions of glucocorticoids in cartilage and bone during health, disease, and steroid therapy. Physiol Rev. 2016;96(2):409-447. 26842265 10.1152/physrev.00011.2015

[4] Shavit E Alavi A Sibbald RG . Vasculitis – what do have to know? A review of literature. Int J Low Extrem Wounds. 2018;17(4):218-226. 30501545 10.1177/1534734618804982

[5] Cooper RL Segal RA Diegelmann R Reynolds AM . Modeling the effects of systemic mediators on the inflammatory phase of wound healing. J Theor Biol. 2015 Feb21;367:86-99. 25446708 10.1016/j.jtbi.2014.11.008

[6] Steingräber AK Schelhaas S Faust A , et al. Molecular imaging reveals time course of matrix metalloproteinase activity in acute cutaneous vasculitis in vivo. Exp Dermatol. 2013 Nov;228(11):730-735. 10.1111/exd.12253 24112050

[7] Watanabe R Maeda T Zhang H , et al. MMP (Matrix metalloproteinase)-9-producing monocytes enable T cells to invade the vessel wall and cause vasculitis. Circ Res. 2018;123(6):700-715. 29970365 10.1161/CIRCRESAHA.118.313206 PMC6202245

[8] Apelqvist J Willy C Fagerdahl AM , et al. Negative pressure wound therapy – overview, challenges and perspectives. J Wound Care. 2017;26(3):S1-S113. 10.12968/jowc.2017.26.Sup3.S1 28345371

[9] Pichler M Larcher L Holzer M , et al. Surgical treatment of pyoderma gangrenosum with negative pressure wound therapy and split thickness skin grafting under adequate immunosuppression is a valuable treatment option: case series of 15 patients. J Am Acad Derm. 2016;74:760-765. 26979359 10.1016/j.jaad.2015.09.009

[10] Eisendle K Thuile T Deluca J Pichler M . Surgical treatment of pyoderma gangrenosum with negative pressure wound therapy and skin grafting, including xenografts: personal experience and comprehensive review on 161 cases. Adv Wound Care. 2020 Jul;9(7):405-425. 10.1089/wound.2020.1160 PMC7307671 32320362

[11] Labler L Mica L Härter L Trenz O Keel M . Influence of V.A.C. Therapy on cytokines and growth factors in traumatic wounds. Zentralbl Chir. 2006;131(Supl 1):S62-S67. 10.1055/s-2006-921511 16575647

[12] Labler L Rancan M Mica L , et al. Vacuum-assisted closure therapy increases local interleukin-8 and vascular endothelial growth factor levels in traumatic wouds. J Trauma. 2009;66:749-757. 19276749 10.1097/TA.0b013e318171971a

[13] Morykwas MJ Argenta LC . Nonsurgical modalities to enhance healing and care of soft tissue wounds. J South Orthop Assoc. 1997;6:279-288. 9434249

[14] Hasan M Teo R Nather A . Negative-pressure wound therapy for management of diabetic wounds: a review of the mechanism of action, clinical applications, and recent developments. Diabetic Foot and Ankle. 2015 Jul 1;6:27618. 26140663 10.3402/dfa.v6.27618 PMC4490797

[15] Wysocki AB Staiano-Coico L Grinnell F . Wound fluid from chronic leg ulcers contains elevated levels of metalloproteinases MMP-2 and MMP-9. J Invest Dermatol. 1993;101:64-68. 8392530 10.1111/1523-1747.ep12359590

[16] Karam RA Rezk NA Abdel Rahman TM Al Saeed M . Effect of negative pressure wound therapy on molecular markers in diabetic foot ulcers. Gene. 2018 Aug 15;667:56-61. 29758297 10.1016/j.gene.2018.05.032

[17] Fraccalvieri M Fierro M Salomone M , et al. Gauze-based negative pressure wound therapy: a valid method to manage pyoderma gangrenosum. Int Wound J. 2014;11(2):164-168. 22891652 10.1111/j.1742-481X.2012.01058.x PMC7950988

[18] Parrado P Cadena M Vergara A , et al. The role of negative pressure wound therapy in the management of hidradenitis suppurative: a case report and literature review. Int Wound J. 2017;14(1):35-39. 26663439 10.1111/iwj.12544 PMC7949800

[19] Pearce Jr FB Richardson KA . Negative pressure wound therapy, staged excision and definitive closure with split-thickness skin graft for axillary hidradenitis suppurativa: a retrospective study. J Wound Care. 2017;26(Sup1):S36-S42. 10.12968/jowc.2017.26.Sup1.S36 28105901

[20] Ohura N Okazaki M Tanba M , et al. Topical negative pressure therapy for para-ileostomal ulceration in a patient with Behcet's Disease. Case report. J Wound Care. 2008;17(2):86-89. 18389833 10.12968/jowc.2008.17.2.28184

[21] Keskitalo S Haapaniemi E Einarsdottir E , et al. Novel TMEM173 mutation and the role of disease modifying alleles. Front Immunol. 2019 Dec 5;10:2770. doi: 10.3389/fimmu.2019.02770. eCollection 2019.PMID: 31866997. 31866997 10.3389/fimmu.2019.02770 PMC6907089

[22] Anghel EL Kim PJ . Negative pressure wound therapy: a comprehensive review of the evidence. Plast Recontstr Surg. 2016;138(3 Suppl):129S-137S. 10.1097/PRS.0000000000002645 27556753

[23] Agarwal P Kukrele R Sharma D . Vacuum assisted closure (VAC)/negative pressure wound therapy (NPWT) for difficult wounds: a review. J Clin Orthop Trauma. 2019;10(5):845-848. 31528055 10.1016/j.jcot.2019.06.015 PMC6739293

[24] Bassetto F Lancerotto L Salmaso R , et al. Histological evolution of chronic wounds under negative pressure therapy. J Plast Reconstr Aesthet Surg. 2012;65:91-99. 21885358 10.1016/j.bjps.2011.08.016

